# Learning Sparse Representations for Fruit-Fly Gene Expression Pattern Image Annotation and Retrieval

**DOI:** 10.1186/1471-2105-13-107

**Published:** 2012-05-23

**Authors:** Lei Yuan, Alexander Woodard, Shuiwang Ji, Yuan Jiang, Zhi-Hua Zhou, Sudhir Kumar, Jieping Ye

**Affiliations:** 1 Center for Evolutionary Medicine and Informatics, The Biodesign Institute, Arizona State University, AZ 85287, USA; 2 Ira A. Fulton Schools of Engineering, Arizona State University, AZ 85287, USA; 3 Department of Computer Science, Old Dominion University, VA 23529, USA; 4 National Key Laboratory for Novel Software Technology, Nanjing University, China; 5 School of Life Sciences, Arizona State University, AZ 85287, USA

## Abstract

**Background:**

Fruit fly embryogenesis is one of the best understood animal development systems, and the spatiotemporal gene expression dynamics in this process are captured by digital images. Analysis of these high-throughput images will provide novel insights into the functions, interactions, and networks of animal genes governing development. To facilitate comparative analysis, web-based interfaces have been developed to conduct image retrieval based on body part keywords and images. Currently, the keyword annotation of spatiotemporal gene expression patterns is conducted manually. However, this manual practice does not scale with the continuously expanding collection of images. In addition, existing image retrieval systems based on the expression patterns may be made more accurate using keywords.

**Results:**

In this article, we adapt advanced data mining and computer vision techniques to address the key challenges in annotating and retrieving fruit fly gene expression pattern images. To boost the performance of image annotation and retrieval, we propose representations integrating spatial information and sparse features, overcoming the limitations of prior schemes.

**Conclusions:**

We perform systematic experimental studies to evaluate the proposed schemes in comparison with current methods. Experimental results indicate that the integration of spatial information and sparse features lead to consistent performance improvement in image annotation, while for the task of retrieval, sparse features alone yields better results.

## Background

**Figure 1:**
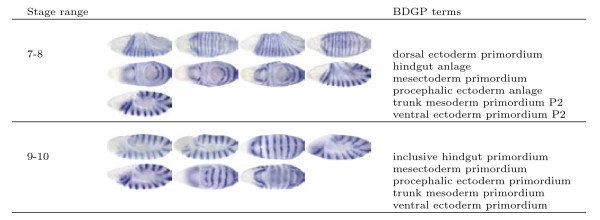
**Sample image groups (all images within a group are from the same stage range and the same gene) and the associated terms from BDGP for the gene *engrailed *in stage ranges 7-8 and 9-10.**

Embryos undergo a temporally ordered differentiation process, starting as basic undifferentiated eggs. Through the process of differentiation, gene expressions take on increasingly complex patterns. Transcriptional regulation of the fruit-fly *Drosophila melanogaster* is one of the best understood examples of the regulatory networks that govern gene expression patterning. An understanding of the regulatory networks responsible for gene patterning in *Drosophila* embryos has been aided by digital images produced via *in situ* hybridization [1-3]. These images document the spatiotemporal dynamics of differentiation found in *Drosophila* embryos. A comparative analysis of these images is beneficial for the understanding of functions and interactions in gene networks [4-14]. To facilitate these discoveries, tools have been developed to searching for images based on keywords that describe embryonic structures [15], and searching for images based on gene expression patterns [13,14]. Images for these tools have been obtained from databases of *Drosophila* embryonic images, e.g. the Berkeley *Drosophila* Genome Project (BDGP), and they are annotated with a controlled vocabulary (CV) [1,2] (Figure 1). The CV terms describe the developmental and anatomical properties of gene expression during embryogenesis [1]. Currently, groups of BDGP images are manually annotated with CV terms. This is done collectively so that not all images in a group necessarily correspond with each CV annotation. The manual nature of these tasks puts an inordinate burden on biologists as the collection of *Drosophila* gene expression patterns are growing rapidly [1]. It is therefore imperative to investigate efficient and effective computational methods to automate this task [16-18].

Image annotation and image retrieval problems have been studied extensively in computer vision and machine learning. However, natural images are the most common subjects of study for image annotation and image retrieval problems; and commonly-used annotation and retrieval techniques may not be effective for our task. For example, unlike most natural images, BDGP images have all been aligned and scaled semi-automatically. The binary feature vector (BFV) representation have been developed correlate pattern similarities between images [13], however the BFV representation is not robust to distortions; there were also some studies which tried to use robust descriptors to represent the BDGP images [19-22], however they have not exploited spatial information. It is desirable to represent images in a way that takes advantage of the spatial properties of image features, while at the same time being robust to image distortions. In our annotation problem, we are interested in collectively annotating groups of images, with each group annotated with multiple CV terms. Previous studies have revealed that ignoring group memberships can be detrimental to annotation performance [19], and formulating the task as learning the function between local input patterns and CV terms lead to significant performance improvement [21].

In this article we propose a novel approach for the automated annotation and retrieval of *Drosophila melanogaster* images. We present an image representation model that takes advantage of the spatial information provided by the BDGP images while at the same time being more robust against distortions. We also take advantage of a state-of-the-art learning model in order to boost the performance of our tasks. Our feature representation framework is inspired by the spatial bag-of-words (BoW) approach for image representation. The BoW approach involves first extracting features from local patches on images. These patches are then quantized to a visual word that has been determined by a pre-computed codebook. Our approach involves extracting these local patches from each image in a group, while maintaining a record of the locations where features are extracted. Thus, our bag-of-words method is essentially a spatial-bag-of-words method. As previous experiments have discovered [16], using only one codebook word to describe a local patch does not capture the slight differences between a word and the actual feature. Therefore, we have adopted a sparse learning framework in order to take advantage of multiple codebook words that show varying levels of similarity to a single feature, leading to a “visual sentence” representation of the image patch.

We have tested our methods on BDGP images from the FlyExpress database (http://www.ﬂyexpress.net). Annotation results from our study show that the spatial-bag-of-words approach consistently outperforms the non-spatial, bag-of-words approach as well as the binary feature vector approach. Results also show that incorporating the sparse learning framework into our representation model further improves performance. While for the image retrieval task, experiments show that utilizing the sparse representation alone is sufficient.

## Methods

In this section, we describe the bag-of-words (BoW) and the sparse learning representations for gene expression pattern image annotation and retrieval.

### The bag-of-words approach

The bag-of-words method was originally used for text classification problems where each document is represented as a feature vector indicating the frequency of each word in the document. Such feature vector representation is used to classify documents into one or more categories. This text categorization approach has been adapted to image analysis [23]. Specifically, images are represented as a collection of “visual words”, based on features extracted from the images [24].

In the BoW approach for image representation, invariant visual features are usually extracted from a subset of images [24] to produce a visual codebook using a clustering algorithm, though a recent study shows that the clustering process is not really essential [25]. Here the cluster centers are considered to be visual words. From this codebook, each feature from an image patch is quantized to the closest visual word in the codebook. A histogram is then created to represent the number of occurrences of each word located in an image. This histogram is a global representation because it only tracks the number of occurrences of each word in an image but not the location of those words, thereby the spatial layout of local image features is not captured. This is considered as one of the major drawbacks of the BoW model [19]. Next, we discuss each step involved in the BoW model when applied to fruit fly images in details.

#### Feature detection

**Figure 2:**
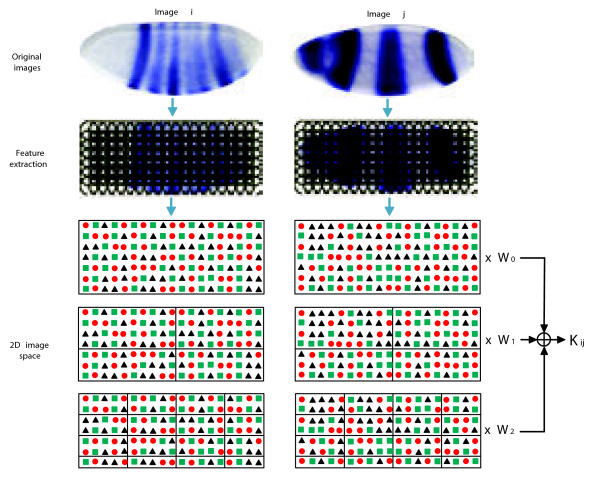
**Illustration of image patch extraction and the three levels of bag-of-words partitioning with weighting factors for the spatial pyramid approach.** After feature description using the overlapping circular patches, three levels of bag-of-words partitioning are shown. The top level of partitioning is just a global bag-of-words representation.

Feature detection involves locating regions in an image to serve as representative boundaries for visual words. We are using images that have been properly scaled and aligned semi-automatically. We use a series of overlapping circles to represent areas where feature information is extracted to construct a single visual word. An example of these overlapping circles is shown in Figure 2. In our experiments, the radius of the patches are set to 16.

#### Feature description

Based on the regions described above, a local feature is extracted from each of the overlapping circle. Because of its robustness against variations in image scale and rotation, we use the scale-invariant feature transform (SIFT) descriptor [26] for representing each local patch. Thus, each image consists of a collection of feature vectors.

#### Codebook generation

The codebook is constructed by obtaining a collection of representative vectors from the extracted features. We use the common generation approach of selecting a subset of images and then using the k-means algorithm to cluster their SIFT feature vectors [27]. The number of cluster centers which represent the visual words can be set manually. For our image annotation and retrieval problem, we have set this number to 2000. The SIFT feature vectors can then be quantized to the closest codebook centers in order to form a visual word representation for each image.

Once the codebook has been created, we can assign codebook words to features extracted from image patches. Formally, assume the number of patches (feature vectors) for a given image is *I* and the size of the codebook is *J*. Define _*eij*_=1 if the ^*ith*^ feature vector is assigned to the ^*jth*^codeword, and 0 otherwise. Then the given image can be represented as $H=\left[h_{1},h_{2},\ldots ,h_{J}\right]$ where

(1) $$h_{j}=\overset{I}{\underset{i=1}{\sum }}e_{\mathrm{ij}}.$$

### The spatial bag-of-words approach

A major limitation of the BoW approach is that the spatial information of local image features is not encoded, as the bag-of-words representation is an un-ordered collection of visual words. A previous study on a bag-of-words approach [19] for automated annotation of *Drosophila* embryo image groups showed encouraging results, and a recent study [21] showed that using spatial information together with visual information is better than using only visual information. We expect the performance can be further improved by taking advantage of the spatial information, i.e., the location where visual words are found within images. Intuitively, the additional spatial information of visual words within images may facilitate the classification of images when the discriminant features are restricted to a certain region, which is the case for our CV terms. This can be implemented by adopting a method similar to the spatial pyramid matching scheme [28].

Our approach for image representation is based on an implementation of the spatial bag-of-words method. Like the BoW method, the spatial BoW method creates a histogram for each image, counting the number of times each word appears in an image. Additionally, the spatial BoW tracks the position where each visual word is located. Therefore, the spatial BoW method benefits from the robustness of the BoW method while also taking advantage of the spatial properties of images.

A spatial bag-of-words is much like a normal bag-of-words except that it is represented by a larger feature vector. While a histogram of an image is represented by a non-spatial bag-of-words, *H*, a spatial bag-of-words consists of multiple non-spatial bags, concatenated. Specifically, for each image with *n* spatial sections, a spatial bag _*Mn*_ can be represented as $M_{n}=\left[H_{1},H_{2},\ldots ,H_{n}\right]$, where each _*Hi*_corresponds to a non-spatial bag-of-words for a particular spatial section. Thus we have *n* bags-of-words from *n* spatial sections on each image that are concatenated to form _*Mn*_. This way, different sections of a spatial vector represent different sections of an image. Our automated annotation representation is created by partitioning feature patches into 3 by 6 sections on each image. This representation creates a multiple of 18 in added dimensionality to a non-spatial representation of the same visual words. For each image group in the study we also create a global bag-of-words representation to test the differences in annotation performance that are seen between the global and the spatial approaches. Figure 2 shows a global bag-of-words representation, a 2 by 2 spatial BoW representation, and a 4 by 4 spatial BoW representation below the circular feature representations of two separate images.

### The sparse spatial representation

The original BoW representation, as applied to image analysis, assigns each feature vector to the closest visual word in the dictionary. Denote the feature vector obtained for a given patch as *y*∈^*Rd*^ and the dictionary matrix as *D*∈^*Rd*×*c*^, in which each column is a centroid (visual word). Then, the assignment of an image patch to a visual word can be written formally as the following optimization problem:

(2) $$\begin{array}{cc} \underset{e}{\min} & \;\frac{1}{2}∥\mathrm{De}-y{∥}_{2}^{2}\\ \text{s.t.} & \;e_{i}\in \{0,1\},\;\overset{c}{\underset{i=1}{\sum }}e_{i}=1 \end{array}$$

Clearly, the constraints enforce that only one element in the solution *e* will be set to one, which corresponds to the visual word most similar to the image patch *y*. In this case, relationships between a feature vector and other visual words are discarded. This would not be a problem if a feature vector is an exact match with the visual word that it is assigned to, as in the case of text classification. However for images, a feature vector may be close to multiple visual words. In such cases, the relationship with the closest word would be overestimated and the relationships with the other similar words would be lost, leading to degenerated representation accuracy.

The sparse approach for BoW representation addresses this problem by assigning feature vectors to multiple visual words simultaneously. We seek to represent the local patch using “visual sentence” with a *set* of “words” instead of a single one. Besides the selection of visual words to form this sentence, we also need to evaluate the “contributions”. A commonly used approach is to formulate this problem as a sparse learning problem, which can be solved by state-of-the-art algorithms.

Mathematically, the generalization from visual word to visual sentence can be done by relaxing the constraint in (2). We construct the representation vector *x*∈^*Rc*^, such that for the ^*ith*^entry, *i*=1,…,*c*, _*xi*_=_*wi*_ when the ^*ith*^ keyword is selected with contribution _*wi*_, and 0 when the keyword is not selected.

In order to make *x* sparse (contains multiple 0 entries), an _*ℓ*1_regularization is imposed, resulting in the following optimization problem:

(3) $$\begin{array}{cc} \underset{x}{\min} & \;∥\mathrm{Dx}-y{∥}_{2}+\lambda |x{|}_{1}\\ \text{s.t.} & \;x_{i}\geq 0,\;i=1,\ldots ,c \end{array}$$

In which _|·|1_ is the _*ℓ*1_ norm and *λ* is a parameter that controls the sparsity. In our experiments, *λ*is fixed to be 0.01. This problem is closely related to *LASSO*[29], and can be solved by many existing software packages, such as SLEP [30].

**Figure 3:**
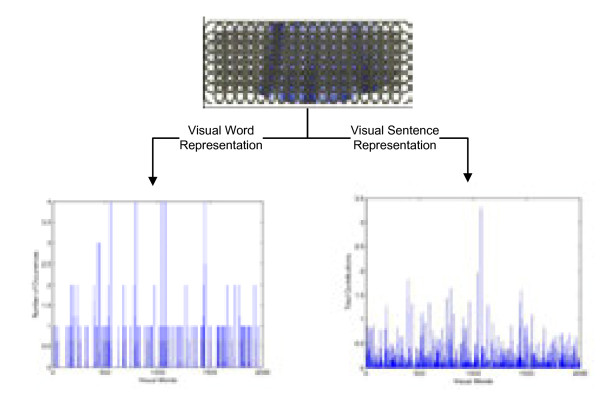
**Different histogram representation obtained for a given image.** The histogram on the left is obtained by assigning each local patch to a single visual word, while the one on the right is obtained by applying the sparse learning formula to select a set of visual words for each patch.

The comparison between “visual word” and “visual sentence” for image representation is illustrated in Figure 3. As shown in the figure, the sparse learning provides more smooth representation.

Integrating the spatial and sparse approaches into the BoW representation model is therefore expected to produce a more accurate description of *Drosophila* images. We have created both sparse and non-sparse versions of both our global and spatial bag-of-words representations, and compare different combinations of approaches for image annotation and retrieval. Detailed performance evaluation can be found in the results section.

## Results and discussion

### Data description

The *Drosophila* gene expression pattern images used in our study are obtained from the FlyExpress database, which contains standardized images obtained from the Berkeley *Drosophila* Genome Project (BDGP). In BDGP, the *Drosophila* embryogenesis is divided into six stage ranges (1-3, 4-6, 7-8, 9-10, 11-12, 13-16). The first stage range is not included in this study because of the small number of CV terms used to describe its images. Images from the remaining stage ranges are annotated separately in their respective groups because the majority of terms are stage range specific. The second through sixth stage ranges consist of 1081, 877, 1072, 2113, and 2816 image groups, respectively. The last two stage ranges contain the largest number of lateral images as well as the highest counts of CV terms.

### Evaluation of annotation performance

We employ the one-against-rest support vector machines (SVM) to annotate the gene expression pattern images, where the SVM builds a decision boundary between image groups that contain a particular term and the remaining image groups. We employ the LIBSVM package [31] and the linear kernel is used. The regularization parameter is set to 1 in all cases. Our proposed method combines both the spatial and sparse approaches and is denoted by SVM_Spatial+Sparse_. We compare our method with those that utilize only sparse, only spatial, or global bag-of-words approaches. These approaches are denoted by SVM_Sparse_, SVM_Spatial_, and SVM_Global_, respectively. The performance comparison of the four representations in terms of AUC and macro F1 scores is summarized in Tables 1 and 2, respectively.

**Table 1:** Comparison of different annotation methods in terms of AUC

**Stage range**	**Number of terms**	**SVM**_**Spatial+Sparse**_	**SVM**_**Sparse**_	**SVM**_**Spatial**_	**SVM**_**Global**_
4-6	10	**.8284 ± .0321**	.8250 ± .0319	.8064 ± .0321	.7984 ± .0320
	20	**.8310 ± .0286 **	.8240 ± .0293	.8046 ± .0292	.7965 ± .0302
	30	**.7982 ± .0408**	.7892 ± .0399	.7777 ± .0400	.7635 ± .0405
7-8	10	**.7808 ± .0285**	.7685 ± .0297	.7567 ± .0301	.7472 ± .0293
	20	**.7734 ± .0431**	.7619 ± .0427	.7444 ± .0496	.7309 ± .0484
9-10	10	**.7917 ± .0260**	.7816 ± .0270	.7652 ± .0264	.7538 ± .0265
	20	**.7971 ± .0335 **	.7829 ± .0344	.7706 ± .0344	.7476 ± .0349
11-12	10	**.8526 ± .0248 **	.8478 ± .0249	.8316 ± .0243	.8257 ± .0240
	20	**.8574 ± .0206**	.8437 ± .0214	.8275 ± .0215	.8091 ± .0228
	30	**.8275 ± .0252**	.8085 ± .0254	.7940 ± .0274	.7673 ± .0268
	40	**.8193 ± .0290 **	.7991 ± .0306	.7810 ± .0304	.7560 ± .0321
	50	**.8084 ± .0351 **	.7894 ± .0363	.7648 ± .0370	.7426 ± .0382
13-16	10	**.8807 ± .0221 **	.8659 ± .0223	.8632 ± .0218	.8398 ± .0225
	20	**.8504 ± .0172**	.8301 ± .0182	.8304 ± .0180	.8001 ± .0177
	30	**.8344 ± .0197**	.8089 ± .0198	.8066 ± .0190	.7713 ± .0198
	40	**.8175 ± .0196**	.7892 ± .0208	.7847 ± .0211	.7496 ± .0223
	50	**.8038 ± .0249**	.7748 ± .0208	.7672 ± .0261	.7340 ± .0271
	60	**.7947 ± .0282**	.7657 ± .0299	.7613 ± .0300	.7281 ± .0310

**Table 2:** Comparison of different annotation methods in terms of macro F1

**Stage range**	**Number of terms**	_**SVMSpatial + Sparse**_	_**SVMSparse**_	_**SVMSpatial**_	_**SVMGlobal**_
4-6	10	**.5224 ± .0407**	.5094 ± .0393	.4926 ± .0414	.4767 ± .0386
	20	**.4454 ± .0461**	.4200 ± .0462	.4141 ± .0459	.3794 ± .0412
	30	**.3459 ± .0593**	.3230 ± .0516	.3153 ± .0565	.2942 ± .0479
7-8	10	**.5372 ± .0343**	.5282 ± .0312	.5131 ± .0329	.5055 ± .0329
	20	**.3653 ± .0517 **	.3603 ± .0538	.3331 ± .0740	.3364 ± .0676
9-10	10	**.5561 ± .0282 **	.5499 ± .0276	.5353 ± .0289	.5267 ± .0260
	20	**.3836 ± .0464**	.3764 ± .0442	.3527 ± .0370	.3429 ± .0342
11-12	10	**.6339 ± .0280**	.6261 ± .0269	.6109 ± .0271	.6060 ± .0257
	20	**.5226 ± .0379**	.4961 ± .0310	.4781 ± .0337	.4508 ± .0290
	30	**.4066 ± .0409**	.3761 ± .0310	.3488 ± .0400	.3373 ± .0300
	40	**.3351 ± .0480**	.3110 ± .0383	.2686 ± .0456	.2762 ± .0358
	50	**.2758 ± .0480 **	.2626 ± .0404	.2343 ± .0434	.2293 ± .0370
13-16	10	**.6506 ± .0297 **	.6310 ± .0272	.6273 ± .0261	.5993 ± .0253
	20	**.5240 ± .0280 **	.4959 ± .0262	.4963 ± .0266	.4580 ± .0245
	30	**.4474 ± .0303**	.4115 ± .0262	.4089 ± .0275	.3692 ± .0243
	40	**.3876 ± .0340**	.3487 ± .0268	.3408 ± .0319	.3071 ± .0252
	50	**.3330 ± .0381**	.2981 ± .0281	.2764 ± .0347	.2607 ± .0263
	60	**.2886 ± .0434**	.2598 ± .0317	.2313 ± .0373	.2255 ± .0287

Since most CV terms are stage-range specific, we annotate the image groups according to their stage ranges separately. The numbers and proportions of positive samples for the 10 most frequent term in each stage range are summarized in Table 3. For each stage range, we begin with the 10 terms that appear most frequently, and then we add additional terms in the order of their frequencies with a step size of 10. This results in different numbers of data sets in each stage range, depending on the total number of CV terms in that stage range. The extracted data sets are randomly partitioned into disjoint training and testing sets using the ratio 1:1 for each term. For each data set, we generate 30 random partitions and the average performance is reported. Because our method models each individual term separately, we can compare the results of our method against the results of the other method on a term-by-term basis. For example, we can compare annotation results of our method with the non-spatial method in stage range 13-16, term by term, where 40 CV terms are used. In this comparison, of the 40 terms being studied, 39 saw an average increased AUC performance and 31 saw average increased F1 Score (F1) performance. Due to space limitation, we will not show each individual term by term comparison. Instead, we show the results for each stage range where various numbers of CV terms are used.

**Table 3:** Number and proportion of postive samples for 10 most frequent terms in each stage range

**Stage Range**	**4-6**	**7-8**	**9-10**	**11-12**	**13-16**
#1	302(27.94%)	390(44.47%)	472(44.03%)	936(44.30%)	1068(37.93%)
#2	259(23.96%)	371(42.30%)	430(40.11%)	882(41.74%)	811(28.80%)
#3	231(21.37%)	358(40.82%)	429(40.02%)	604(28.58%)	791(28.09%)
#4	216(19.98%)	342(39.00%)	413(38.53%)	568(26.88%)	642(22.80%)
#5	199(18.41%)	273(31.13%)	306(28.54%)	554(26.22%)	564(20.03%)
#6	195(18.04%)	241(27.48%)	249(23.23%)	475(22.48%)	517(18.36%)
#7	107(9.90%)	162(18.47%)	224(20.90%)	284(13.44%)	492(17.47%)
#8	91(8.42%)	145(16.53%)	215(20.06%)	263(12.45%)	389(13.81%)
#9	90(8.33%)	103(11.74%)	128(11.94%)	261(12.35%)	353(12.54%)
#10	87(8.05%)	84(9.58%)	103(9.61%)	232(10.98%)	324(11.51%)

Table 1 shows a comparison of AUC results for all four methods discussed. The best results for each case are highlighted in bold. The results show that both the spatial and the sparse methods consistently outperform the non-spatial method in terms of average AUC. The results also show that combining both sparse and spatial approaches outperforms any of the other three methods. The results indicate that the sparse approach offers improved performance over the spatial approach for the earlier stage ranges, and that the two approaches are comparable for the last stage range. The poorer performance of the spatial approach for the earlier stages may have been due to the less developed embryonic structures found earlier in embryogenesis. Combining the spatial and sparse approaches resulted in the best results, particularly in the later stage ranges.

Table 2 shows a similar type of comparison as in Table 1. The only difference is that F1 score is used as a comparison measure instead of AUC. We observe a similar trend: both the spatial and sparse methods outperform the global approach; the sparse approach performs slightly better than the spatial approach in the earlier stages, and they achieve similar performance during the last stage. Again, we can observe that combining the sparse and spatial approaches generates better results than using sparse or spatial information alone.

**Figure 4:**
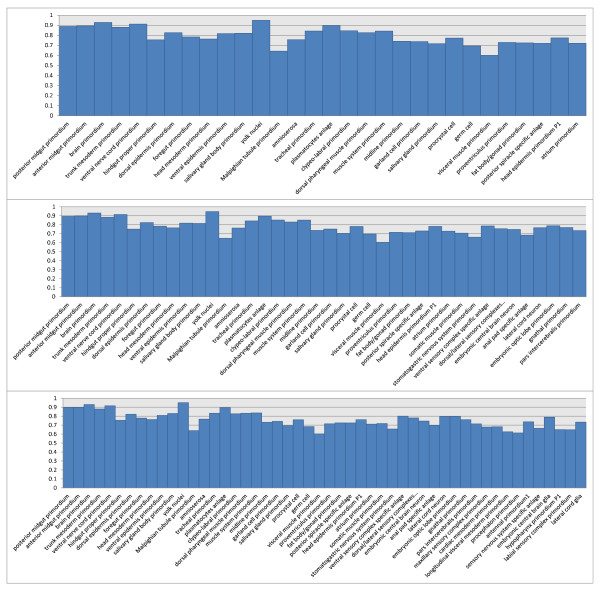
**The AUC of individual terms on three data sets from stage range 11-12.** The three figures, from top to down, show the performance with 30, 40, and 50 terms, respectively.

**Figure 5:**
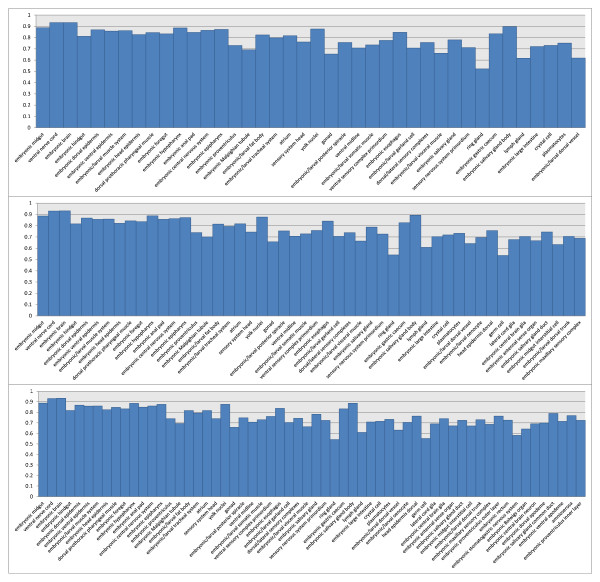
**The AUC of individual terms on three data sets from stage range 13-16.** The three figures, from top to down, show the performance with 40, 50, and 60 terms, respectively.

We have observed that there were significant differences in performance increases between earlier stage ranges where Drosophila embryos were less developed and later stage ranges where embryos were more developed. We also observe that there are certain terms that benefit far greater from a spatial bag-of-words approach than other terms. For example, *mesectoderm anlage in statu nascendi*, *central brain anlage*, *crystal cell specific anlage*, *hypopharynx primordium P2*, *procrystal cell*, and *crystal cell* are all stage dependent terms that showed the most dramatic increases in annotation performance. These increases in performance were consistent across multiple stage range tests, where the number of terms being annotated varied. There are also a number of terms such as *pole cell*, *mesectoderm primordium*, *foregut primordium*, *germ cell*, *embryonic central brain neuron*, *embryonic central brain glia*, and *lateral cord glia* that showed good performance across multiple stage ranges, where various numbers of CV terms were annotated. We included detailed performance evaluation of individual terms in 6 different data sets in Figures 4 and 5.

There are pioneering works on constructing feature representations for Drosophlia gene expression image annotation. Zhou et al. [32] applied multi-resolution 2D wavelet discrete transform followed by min-Redundancy max-Relevance feature selection. Puniyani et al. [12] proposed an automatic system named “SPEX^2^” that performs pattern extraction using Markov random field and further extracts features using the SIFT descriptor and singular value decomposition. Using the top 10 most frequent terms [12] in the BDGP data set, Zhou’s system achieved an average F1 score of about **0.35**, while Puniyani’s method achieved about **0.45**. For comparison purposes, we extract the individual F1-scores for the same terms. Our Sparse + Spatial representation yields an average F1-score of **0.64**, which outperforms both methods.

#### Comparison of different classifiers

Since the main focus of this section is to demonstrate the performance of various image representations, we fix our classifier to be SVM with linear kernel for its effectiveness in high-dimensional data. However, it will also be interesting to investigate how different classifiers perform in this task. As an illustrative example, we use stage range 11-12 with sparse representation and test the classification performance of three different classifiers including SVM, logistic regression and ridge regression. The performance in terms of sensitivity and specificity is reported in Table 4. For all three methods, we apply 4-fold cross validation for parameter selection. As we can see in Table 4, the three classifiers achieve comparable overall performance, and SVM achieves slightly higher sensitivity.

**Table 4:** Performance evaluation in terms of sensitivity and specificity

	**Sensitivity**			**Specificity**		
**# of terms**	**SVM**	**Logistic**	**Ridge**	**SVM**	**Logistic**	**Ridge**
10	0.6211 ± 0.020	0.6267 ± 0.023	0.6307 ± 0.020	0.8520 ± 0.012	0.8460 ± 0.012	0.8323 ± 0.012
20	0.4633 ± 0.020	0.4483 ± 0.020	0.4441 ± 0.017	0.9252 ± 0.006	0.9354 ± 0.006	0.9309 ± 0.006
30	0.3306 ± 0.025	0.3154 ± 0.023	0.3038 ± 0.019	0.9523 ± 0.004	0.9566 ± 0.004	0.9573 ± 0.004
40	0.2549 ± 0.015	0.2424 ± 0.014	0.2320 ± 0.012	0.9628 ± 0.003	0.9677 ± 0.003	0.9668 ± 0.003
50	0.2032 ± 0.012	0.1974 ± 0.011	0.1910 ± 0.012	0.9724 ± 0.003	0.9732 ± 0.003	0.9723 ± 0.003

Sparse feature is used and the classification performance on stage range 11-12 is reported. Three different classifiers are applied for comparison, namely, SVM with linear kernel (SVM), logistic regression (Logistic) and ridge regression (Ridge).

#### Performance of over-sampling

As we can see in Tables 2 and 4, when the number of labels is large, the average sensitivity as well as F1 score is quite low. This is due to the dramatic lack of positive samples for some labels. For example, in stage range 11-12, when we use 50 labels, the proportion of positive samples in these 50 labels can be as low as 0.8%. In this subsection, we present some preliminary results on tackling this problem with over-sampling.

The over-sampling method works as follows. Before training a classifier for a particular label, we first do random sampling on the positive samples with replacement, so that the number of positive samples is equal to the negative ones. Then, we train the classifier using the balanced samples. We test this method using the same setting as in the previous subsection, and the classification performance is presented in Table 5. As we can see in Tables 4 and 5, the over-sampling method provides promising improvements in this example, especially when the number of labels is large. For example, when using the logistic regression on annotating 50 labels, the over-sampling improves sensitivity from 0.2 to 0.36. Exploring methods such as over-sampling to further improve the classification performance will be an interesting future direction.

**Table 5:** Performance evaluation of the over-sampling method in terms of sensitivity and specificity

	**Sensitivity**			**Specificity**		
**# of terms**	**SVM**	**Logistic**	**Ridge**	**SVM**	**Logistic**	**Ridge**
10	0.6544 ± 0.026	0.6494 ± 0.027	0.6288 ± 0.020	0.8577 ± 0.012	0.8580 ± 0.012	0.8586 ± 0.015
20	0.4796 ± 0.020	0.5051 ± 0.020	0.4736 ± 0.019	0.9260 ± 0.006	0.9235 ± 0.006	0.9284 ± 0.007
30	0.3487 ± 0.023	0.3741 ± 0.024	0.3643 ± 0.035	0.9484 ± 0.004	0.9447 ± 0.005	0.9265 ± 0.032
40	0.2831 ± 0.017	0.3291 ± 0.018	0.2791 ± 0.026	0.9563 ± 0.004	0.9385 ± 0.004	0.9406 ± 0.023
50	0.2958 ± 0.024	0.3582 ± 0.025	0.2214 ± 0.025	0.9466 ± 0.006	0.9089 ± 0.010	0.9569 ± 0.023

Sparse feature is used and the classification performance on stage range 11-12 is reported. Three different classifiers are applied for comparison, namely, SVM with linear kernel (SVM), logistic regression (Logistic) and ridge regression (Ridge).

### Evaluation of retrieval performance

**Figure 6:**
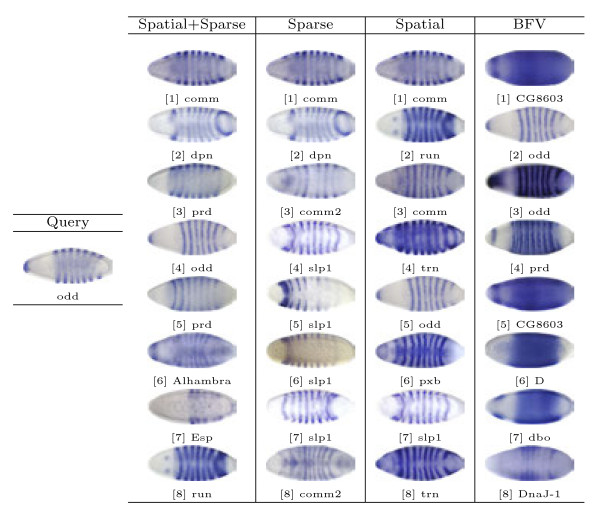
Retrieval results for query image ID insitu21869 with the dorsal view in stage range 4-6.

**Figure 7:**
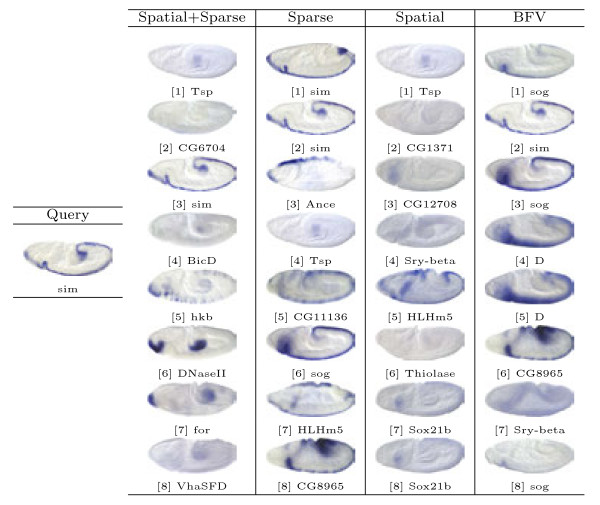
Retrieval results for query image ID insitu22067 with the lateral view in stage range 7-8.

**Figure 8:**
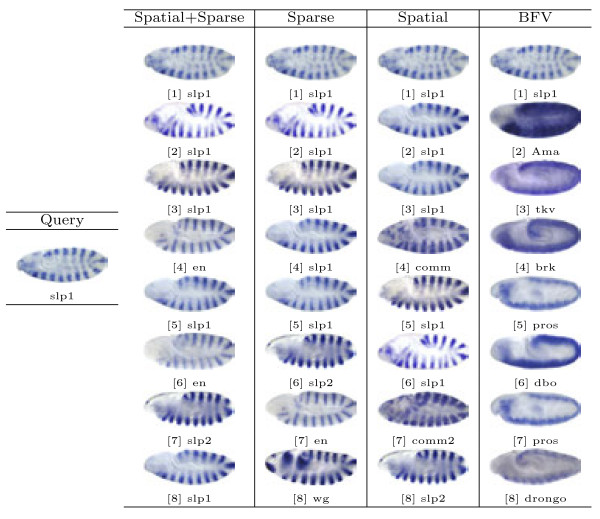
Retrieval results for query image ID insitu16633 with the lateral view in stage range 9-10.

**Figure 9:**
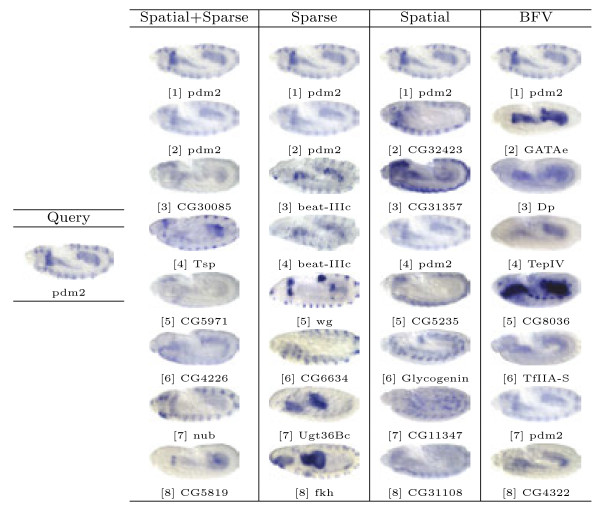
Retrieval results for query image ID insitu21912 with the lateral view in stage range 11-12.

**Figure 10:**
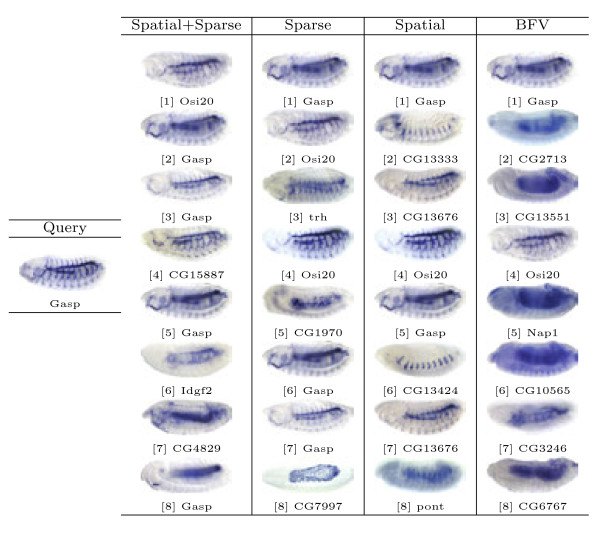
Retrieval results for query image ID insitu23837 with the lateral view in stage range 13-16.

Based on the proposed image representations, we obtain the pair-wise similarity for every two images in the database, which can be used for image retrieval. In our study, the representative images for different views and stage ranges from the well-known Interactive Fly website^a^ are used as queries. Then, for a given method and a query image, we select 8 images with the highest similarity values to obtain a set of query results. Note that the query images are removed from the results since they are always the one with highest similarity. Sample query results from different views and stage ranges are presented in Figures 6, 7, 8, 9 and 10.

First, we will compare different methods by visually inspecting the images retrieved for each query. The first conclusion we can draw from the figures is that the methods based on the bag-of-words (the first three columns) generally outperform the one that utilizes the binary representation only. For example, for the stripe patterns such as those in Figures 6 and 8, the BFV method retrieves less than 4 similar images in its top 8 matches, and in Figure 6, even the best match looks quite different from the query image. Also, we can observe that among the three proposed methods, the sparse representations generally yield more satisfactory results, particularly when the layout of the pattern is subtle, such as the ones in Figure 7.

We also give brief interpretations of the retrieved images by analyzing the functions of the corresponding genes in the biological process annotated in the gene ontology^b^. Figure 6 shows a stripe pattern expressed by gene *odd*, obtained from the dorsal view, in stage range 4-6. *odd* is in charge of the periodic partitioning. The retrieved genes *prd* and *slp1* are about periodic partitioning and blastoderm segmentation, respectively. Both of them are closely related to the query gene. We also observe that several other retrieved genes, such as *comm, comm2, run, trn* and *Alhambra*, are not directly related to the segmentation process. However, they are all involved in the development of the nerve system. It will be interesting to examine how these two functions are related.

Figure 8 shows a pattern expressed by gene *slp1*, during stage range 9-10. As we can see, all of the three “visual sentence” based approaches retrieved 6 images with *slp1* expressed. The rest of the genes retrieved, such as *slp2* which is involved in periodic partitioning, and *en* which is associated with the head segmentation process, are all closely connected to the blastoderm segmentation controlled by *slp1*.

Figure 9 is taken from the lateral view, during stage range 11-12. The corresponding gene *pdm2* is linked to the nervous system development. We can observe that our proposed method with the “visual sentence” concept returns 2 images with the same gene as the top query results. The gene *nub* takes part in the fate determination of ganglion mother cell, neuroblast. *beat-IIIc* and *wg* are related to the formation of synapse and endoderm, respectively.

Figure 10 illustrates a pattern expressed by gene *Gasp*, during stage range 13-16, taken from the lateral view. The spatial and sparse representation retrieves 4 images with the same gene, compared to 2 images by spatial BoW and 1 image obtained by BFV. *Gasp* as well as *CG13676* is involved in the chitin metabolic process. Another gene, *Idgf2*, which is related to the chitin catabolic process, is also closely related. The *trh* gene, which affects the epithelial cell fate determination and open tracheal system, is also related because chitin regulates epithelial tube morphogenesis; in addition to its classical role, protecting mature epithelia.

## Conclusions

This article presents computational methods for annotating *Drosophila* gene expression pattern images, and identifying similar images based on gene patterning. In both tasks, images are represented as bags-of-words. The size of the bags is determined by the spatial properties of a representation. For both applications, a sparse learning framework was used. Results on the FlyExpress database indicate that the proposed annotation method outperforms the non-sparse, non-spatial bag-of-words method, as well as approaches that would use either a sparse or spatial framework.

In our study, the bag-of-words representations were created by partitioning image features with local feature patches. Terms that saw the greatest increases in annotation accuracy may only reside in specific regions of *Drosophila* embryos during a given stage of development. one promising direction is to create local bag-of-words from these regions in order to eliminate some of the noise created by other unrelated regions, when searching for specific embryonic structures. This technique is commonly referred to as region of interest (ROI). We plan to explore this in the future.

## Endnotes

^a^http://www.sdbonline.org/ﬂy/aimain/1aahome.htm^b^http://www.geneontology.org/

## Authors’ contributions

All authors analyzed the results and wrote the manuscript. SJ and JY conceived the project and designed the methodology. AW and LY implemented the programs and drafted the manuscript. SJ, YJ, Z, SK, and JY supervised the project and guided the implementation. All authors have read and approved the final manuscript.
